# Investigating the relationship between periodontitis and specific memory processes in the search for cognitive markers of Alzheimer’s disease risk

**DOI:** 10.1038/s41598-023-38674-w

**Published:** 2023-07-18

**Authors:** Michał Wereszczyński, Aleksandra Śmigiel, Iwona Tomaszewska, Agnieszka Niedźwieńska

**Affiliations:** 1 Department of Psychology, Jagiellonian University, Kraków, Poland; 2 Doctoral School in the Social Sciences - Jagiellonian University, Kraków, Poland; 3 Stomatologia Tomaszewska” Dental Office, Kraków, Poland; 4 Centre of Innovative Medical Education, Collegium Medicum, Jagiellonian University, Kraków, Poland

**Keywords:** Human behaviour, Geriatrics, Dementia

## Abstract

The spontaneous retrieval deficit (SRD) hypothesis argues that individuals in the preclinical stages of Alzheimer’s disease (AD) are particularly impaired in spontaneous retrieval, which manifests in reduced mind-wandering. Our main purpose was to provide novel evidence to support the SRD hypothesis by investigating, for the first time, the relationship between mind-wandering and periodontitis, the latter being the risk factor for AD. The second objective was to address the lack of deeper understanding of the relationship between oral health and specific cognitive abilities by investigating whether periodontitis would be primarily associated with memory. Sixty community-dwelling dementia-free older adults completed neuropsychological tests that focused on various cognitive abilities and a computerised task, during which mind-wandering was evaluated. Periodontal health was assessed subjectively, and through an oral examination by a qualified dentist that focused on visible periodontitis-related changes in gingival tissues and the number of periodontitis bacteria. In line with our predictions, objective and subjective symptoms of poorer periodontal health were associated with less mind-wandering, providing further support for the SRD hypothesis. Again in line with predictions, poorer periodontal health was associated with worse episodic memory, with no relationship between periodontitis and the measure targeting various cognitive abilities, from which memory was excluded.

## Introduction

Alzheimer’s disease (AD) is a major cause of dementia and one of the leading causes of death in the elderly age group^1^. Cerebral pathological changes associated with the disease can precede clinical symptoms by up to 20 years^2^. Given the lack of an effective cure, research has progressively focused on identifying people at risk of developing AD among which early intervention can delay and even prevent the emergence of clinical syndrome^3,4^.

Recently, a novel hypothesis has been formulated that argues that spontaneous (i.e., unintentional and effortless) retrieval processes, which are relatively well preserved in healthy ageing^5–7^**,** will be significantly compromised during the prodromal and early stages of AD^8^. The spontaneous retrieval deficit (SRD) hypothesis is highly counterintuitive because it challenges current theories of cognitive ageing^9,10^, which predict that both typical and atypical ageing predominantly impair performance in difficult cognitive tasks dependent on deliberate and strategic processes. However, the hypothesis is based on the results of neuroimaging studies showing that, during the etiology of AD, the neurological structures of the default mode network (DMN) tend to degenerate much earlier than other parts of the central nervous system (see^8^ for a review of evidence).

The first signs of neuropathological changes within AD tend to occur in the posterior parts of the cortex, with the anterior and dorsolateral prefrontal cortex remaining relatively intact^11,12^, resulting in disproportionate temporoparietal atrophy in the early stages of the disease^13,14^. The pathology involves the accumulation of tau-positive neurofibrillary tangles in the structures of the medial temporal lobe, spreading from the entorhinal cortex to the hippocampus^15^, and the formation of β-amyloid plaques in the medial prefrontal and posteromedial cortices, especially in the posterior cingulate cortex and adjacent areas^16,17^. These neuropathological processes, especially β-amyloid accumulation, may progress for decades before the onset of dementia^2^.

Importantly, the posterior cingulate cortex, the medial temporal lobe, and the medial prefrontal cortex form the key hubs of DMN^18,19^. DMN activity has traditionally been associated with mind-wandering, which involves spontaneous shifts of attention from the external world to one’s inner thoughts^20,21^. Links between mind-wandering and increased DMN activity have also been demonstrated in several fMRI studies (see^8^ for a review of evidence). Mind-wandering shares similar characteristics with several other phenomena of spontaneous cognitions such as, for example, involuntary autobiographical memories^22,23^ or those aspects of prospective memory that involve effortlessly remembering previously intended actions in response to a particular target event^24,25^. What these phenomena share with mind-wandering episodes is that thoughts and memories come to mind spontaneously and effortlessly, without any deliberate intention to think about them.

Spontaneous retrieval, when measured during simple cognitive tasks, can provide a promising alternative to the neuropsychological tests of episodic memory currently used to detect an increased risk of AD. Since spontaneous retrieval is not affected by deliberate strategies to enhance recall and, during examination, participants are not aware of what exactly is being measured, such tasks may have a particular advantage for testing highly functioning dementia-free older adults. In such groups, high education and well-developed learning strategies can mask very early signs of cognitive change in standard neuropsychological tests.

A few recent behavioural studies support the SRD hypothesis by showing the deficit of spontaneous retrieval in amnestic Mild Cognitive Impairment (aMCI), which is the prodromal stage of AD, and in early stages of AD^26–29^. Amnestic MCI manifests itself in subjective and objective memory deficits, as evidenced by neuropsychological tests that measure episodic memory, without the loss of functional independence characteristic of AD^3^. Behavioural evidence of spontaneous retrieval deficits in the prodromal and early stages of AD has been found in prospective memory^27,30,31^ and mind-wandering^26,28,29^ (details of behavioural evidence from prospective memory studies in^8^). In mind-wandering studies, thought probes are used alongside very easy cognitive tasks, e.g., deciding whether lines presented on the computer screen are horizontal or vertical, or whether presented pictures show natural or man-made objects. In each probe, participants are asked whether they had any thought at the time they were stopped, and if so, whether it was related to the ongoing task, and if it was spontaneous or deliberate. Participants with aMCI reported significantly less mind-wandering, as measured by the number of spontaneous task-unrelated thoughts, than healthy older adults^28,29^. At the same time, the aMCI groups did not outperform healthy older adults on ongoing tasks, and they continued to mind-wander less after controlling for the ongoing task performance. Studies involving the aMCI groups also suggested that the type of thoughts analysed, i.e., whether related to environmental stimuli or not, and whether oriented toward the past, present, or future, can matter for how strongly the spontaneous retrieval deficit manifests itself^28,29^.

The primary objective of our study was to provide more evidence to support the SRD hypothesis by investigating, for the first time, the relationship between mind-wandering and one of the factors that appears to increase the risk of cognitive decline and AD, namely periodontitis (see meta-analyses of longitudinal studies on periodontitis as a risk factor for AD^32,33^)*.* We expected that cognitively healthy older adults (i.e., without cognitive deficits related to dementia or other diseases), but with poorer periodontal health, would show reduced spontaneous retrieval, that is, less mind-wandering. Although the relationship between mind-wandering and periodontitis has not been investigated for far, the results of only two studies on periodontal health and prospective memory can be interpreted in terms of the link between periodontitis and spontaneous retrieval deficits^34,35^. In both studies, poorer periodontal health was associated with poorer event-based prospective memory, but not with the other type of prospective memory (time-based)^34^. The analyses of prospective memory mechanisms^36,37^ suggest that performance in event-based tasks can be based on spontaneous retrieval of intended actions, whereas time-based tasks require strategic and effortful retrieval.

Several pathophysiological mechanisms could explain the negative impact of poor oral health on cognitive function and its role in the development of AD. For example, Dominy et al.^38^ have identified *Porphyromonas gingivalis*, an organism associated with chronic periodontitis, in the brain of patients with Alzheimer’s disease, and suggested that this microorganism may play a vital role in the disease pathway. A widespread view is that the relationship between AD progression and infection with periodontitis bacteria may occur through the inflammatory pathway, where people with periodontitis are systemically affected by chronic oral inflammation^39–41^. According to the most established model of Kamer et. al.^41–43^, periodontitis pathogens or pro-inflammatory cytokines produced in the immune response to infection can enter the central nervous system (through systemic circulation or through the neural pathways) where they may trigger an immune reaction of glia cells. The model holds that chronic inflammation within the brain stimulates glia cells to produce β-amyloid, tau-fibre and pro-inflammatory molecules that, by inducing autoimmune reaction, cause neurodegeneration. Claims about the triggering role of peripheral gum inflammation in AD etiology are supported by numerous studies that show a significant relationship between AD and the level of periodontitis antibodies^43–45^. Diet and nutrition could also explain the link. Older people with tooth loss, particularly edentulism, could suffer from impaired masticatory function and consequently poor nutritional status^46^.

A straightforward argument can also be presented for the opposite direction of causality behind the link between oral health and cognitive function, i.e., how cognitive decline could negatively impact oral health through behavioural changes such as reduced attention to oral hygiene or inadequate use of dental health services. However, a recent large-scale longitudinal study^47^ shows that the relations between oral health and cognitive function are indeed bidirectional.

The second goal of our study was to investigate whether periodontitis is primarily associated with memory ability. It has recently been suggested^34,48^ that existing studies on the relationship between oral health and cognitive function provide little insight into the nature of cognitive difficulties that are associated with poor periodontal health. This is due to the fact that many of these studies used brief screening measures of general cognitive function rather than comprehensive tests that target specific cognitive abilities. The distinction between specific cognitive abilities is critical to inform the mechanisms underlying the association between oral health and cognition. For example, individuals in early stages of AD show deficits primarily in episodic memory, while individuals in early stages of other types of dementia (i.e., cardio-vascular, frontal–temporal, or lewy bodies) show deficits mainly within other cognitive domains^49–51^. Therefore, if episodic memory is primarily related to periodontal health, this may suggest that we need to look for explanations that associate periodontitis with AD, rather than other types of dementia. A recent systematic review of Nangle et al.^48^ suggests that memory is in fact one of few cognitive abilities that may be specifically related to periodontal health.

To achieve these goals, we performed a neuropsychological assessment that targeted various cognitive abilities in a group of 60 dementia-free community-dwelling older adults. The assessment included, among other tests, a comprehensive episodic memory test (California Verbal Learning Test)^52^. Periodontal health was subjectively evaluated with the list of symptoms, and then objectively through an oral examination conducted by a qualified dentist in a specialist dental clinic. The objective evaluation involved calculating: (1) the community periodontal index of treatment needs (CPITN), and (2) the number and type of periodontitis pathogens present within the periodontium. Mind-wandering was evaluated during a very easy Man-made/Natural Task^5,29^. Participants were repeatedly stopped to report whether they had any thought at the time they were stopped and, if so, if it was related to the Man-made/Natural Task, and whether it was spontaneous or deliberate.

In line with the SRD hypothesis, we expected that poorer periodontal health would be associated with less mind-wandering, that is, less spontaneous, task-unrelated thoughts. Based on the results of a systematic review on how oral health can be related to specific cognitive abilities in older adults^48^, and data suggesting that periodontitis increases the risk of AD rather than the risk of other types of dementia^44,45^, we expected that poorer periodontal health would be associated with a lower performance on the episodic memory test, rather than on the measure targeting various cognitive abilities (Addenbrooke’s Cognitive Examination III), from which memory was excluded.

The novelty of our approach was twofold. First, it provided a new way of testing the SRD hypothesis, by going beyond the spontaneous retrieval deficits in aMCI and early stages of AD, and measuring spontaneous retrieval in cognitively healthy older adults in relation to the risk factor for AD. Second, it addressed the concern of a lack of deeper insight into the relationship between oral health and cognitive function, by investigating links between oral health and the specific cognitive domain (memory) and the specific cognitive process (spontaneous retrieval).

## Results

The alpha level adopted to determine the significance of the results was set at 0.05. Pearson’s coefficients were used to measure correlations between periodontitis status and mind-wandering, and then periodontitis status and tests targeting episodic memory and other cognitive abilities. The strength of the correlations was interpreted according to Cohen’s criteria (0.1 = small; 0.3 = moderate; 0.5 = large)^53^. The correlations and t-test results were controlled for multiple comparisons using the Benjamini–Hochberg procedure^54^ (False Discovery Rate = 0.25). Only those correlations and t-test results that remained significant after the correction are reported, except for tables that present all correlations. When significant associations were established, hierarchical multiple regression models were performed to adjust for background factors that included age, years of education, and MMSE scores.

### Periodontitis status and mind-wandering: testing the SRD hypothesis

There have been 261 valid sextants in the sample, with 10% of sextants with CPITN 1, 24% with CPITN 2, 46% with CPITN 3, 20% with CPITN 4, and only one sextant with CPITN 0, excluded from the analyses. Periodontitis pathogens were detected in the entire sample with *Capnocytophaga gingivalis* in 93% of the participants, *Tannerella forsythia* in 86%, *Treponema denticola* in 75%, *Peptostrep. (Micromonas) micros* in 71%, *Porphyromonas gingivalis* in 50%, and *Fusobacterium nucleatum* in 41%. Three pathogens were very rare in the sample, thus reducing the number of participants for correlation analyses to 12 or less (*Eubacterium nodatum* detected in 20% of the participants, *Aggregatibacter actinomycetemcomitans* 18%, and *Prevotella intermedia* 13%). Therefore, these three pathogens were excluded from the analyses (see Table 1 for the means of all CPITN indices, the number of periodontitis pathogens, and the number of subjectively evaluated periodontitis symptoms).

**Table 1:** Summary of mean results for demographic variables, mind-wandering, neuropsychological tests, and oral examinations.

Variable	Mean	SD
Age	72.517	4.152
Education (years)	16.083	2.757
Mind-wandering: spontaneous task-unrelated thoughts		
Picture-related: present	6.333	5.190
Past	5.017	5.000
Future	1.783	2.471
Picture-unrelated: present	0.417	0.979
Past	0.017	0.129
Future	0.217	0.865
Geriatric depression scale 15	2.467	2.281
MMSE	28.117	1.519
Addenbrooke’s Cognitive Examination-III		
Attention	17.400	0.785
Fluency	11.850	1.938
Visuospatial functions	15.750	0.541
Language	25.783	0.454
Total (excluding memory)	70.783	2.484
California Verabal Learning Test		
Trial 5: free recall	11.150	2.385
Trial 5: intrusion errors	0.267	1.205
Trials 1–5: free recall	47.717	10.064
Trials 1–5: intrusion errors	1.333	2.398
Short delay free recall	9.33	3.317
Short delay free recall intrusion errors	0.25	0.541
Long delay free recall	9.90	3.203
Long delay free recall intrusion errors	0.57	1.267
Community periodontal index of treatment needs (CPITN) scores		
Sum of sextants with CPITN 1^1^	.43	.810
Sum of sextants with CPITN 2	1.03	1.235
Sum of sextants with CPTIN 3	2.02	1.479
Sum of sextants with CPTIN 4	0.85	1.162
Mean CPITN^2^	2.781	0.618
Highest CPITN^3^	3.42	0.671
The number of periodontitis symptoms evaluated subjectively	3.950	2.727
The number of periodontitis pathogens		
*Porphyromonas gingivalis*	900,595,5	2,310,386
*Treponema denticola*	204,062	439,252,2
*Tannerella forsythia*	125,645,2	283,586,5
*Peptostrep. (Micromonas) micros*	6108,333	13,017,13
*Fusobacterium nucleatum*	4891,167	14,331,26
*Capnocytophaga gingivalis*	32,865,33	45,195,72

^1^There was only one sextant rated CPITN 0 across all participants, so sextans with code 0 are not included.

^2^Sum of CPITN codes divided by number of valid sextants.

^3^The highest CPITN code among valid sextants.

Based on the participants’ responses to whether they had any thought in a thought probe, and if so, if it was related to the experience of doing the Man-made/Natural Task, and whether it was spontaneous or deliberate, we calculated the number of spontaneous task-unrelated thoughts (the amount of mind-wandering). In line with the literature, these thoughts were further divided into: (a) picture-related vs. picture-unrelated (i.e., whether related to pictures presented as part of the Man-made/Natural Task or not), (b) present versus past versus future oriented (see Table 1 for the mean number of each type of spontaneous task-unrelated thoughts).

Table 2 shows zero-order correlations between periodontitis status and mind-wandering measures. In line with our predictions, periodontitis status, measured objectively and subjectively, was significantly related to several types of spontaneous task-unrelated thoughts during the Man-made/Natural Task, and for each of these types of thoughts, poorer periodontal health was related to fewer mind-wandering, and better periodontal health was related to more mind-wandering. For objective measures of periodontitis status, there has been a moderate positive correlation between the number of sextants with CPITN 1 (the more sextants with CPITN 1, the better periodontal health) and the number of spontaneous, task-unrelated thoughts that were picture-unrelated and present-oriented. This type of spontaneous task-unrelated thoughts was also negatively related to the mean CPITN, which was the sum of CPITN codes divided by the number of valid sextants (small correlation), and the highest CPITN code among valid sextants (correlation close to moderate). For the subjective measure of oral health, the number of periodontitis symptoms reported by the participants was negatively associated with the number of spontaneous task-unrelated thoughts that were picture-related and oriented either toward the future (moderate correlation) or past (small correlation).

**Table 2:** Testing the relationship between poorer periodontal health and fewer mind-wandering with zero-order correlations between community periodontal index of treatment needs (CPITN) scores and mind-wandering measures.

	Mind-wandering: spontaneous task-unrelated thoughts					
	Picture-related			Picture-unrelated		
	Present	Past	Future	Present	Past	Future
Community periodontal index of treatment needs (CPITN) scores						
Sum of sextants with CPITN 1^1^	.078	.027	− .045	.324*	− .07	.081
Sum of sextants with CPITN 2	− .137	− .11	− .053	− .138	− .11	− .134
Sum of sextants with CPTIN 3	.194	0	.117	− .052	.087	.13
Sum of sextants with CPTIN 4	.028	− .026	− .088	− .168	.243	− .085
Mean CPITN^2^	.105	.009	− .03	− .277*	.152	− .086
Highest CPITN^3^	.037	− .083	− .047	− .294*	.114	− .1
The number of periodontitis symptoms evaluated subjectively	.056	− .261*	− .351**	− .036	.002	− .11

Values that remained statistical significant after Benjamini–Hochberg correction in bold.

^1^The higher the number of sextans with CPITN 1 to 2, the better the gingival health; the higher the number of sextans with CPITN 3 to 4, the poorer the gingival health.

^2^Sum of CPITN codes divided by number of valid sextants; a higher score indicates poorer gingival health.

^3^The highest CPITN code among valid sextants; a higher score indicates poorer gingival health.

*p < 0.05, **p < 0.01.

Table 3 shows the results of hierarchical regression analyses predicting mind-wandering from background variables (age, years of education, and MMSE scores) and CPITN scores. The amount of mind-wandering, as measured by the number of spontaneous, task-unrelated thoughts that were picture-unrelated and present-oriented, remained positively associated with the number of CPITN 1 sextants (*β* = 0.348; *p* < 0.05), and negatively associated with the mean CPITN (*β* = − 0.201; *p* < 0.05) and the highest CPTN (*β* = − 0.282; *p* < 0.05; Table 3). For each of these measures of periodontitis status, the addition of the periodontitis status score as a second step in the regression model contributed uniquely and significantly (see Table 3). Table 3 also shows the results of hierarchical regression analyses that predict mind-wandering from background variables (age, years of education, and MMSE scores) and the number of periodontitis symptoms chosen by the participant on the list of symptoms. The number of symptoms remained negatively associated with the amount of mind-wandering that was picture-related and future-oriented (*β* = − 0.316; *p* < 0.05), and this number contributed significantly when added as a second step in the regression model (Table 3). However, the number of symptoms was no longer a significant predictor of mind-wandering that was picture-related and oriented toward past.

**Table 3:** Hierarchical multiple regression analyses predicting mind-wandering from age, education, MMSE scores and CPITN scores^1^.

	Mind-wandering picture-unrelated about present^2^			Mind-wandering picture-unrelated about present^2^			Mind-wandering picture-unrelated about present^2^			Mind-wandering picture-related about past^3^		Mind-wandering picture-related about future^4^	
	ΔR^2^	β		ΔR^2^	β		ΔR^2^	β		ΔR^2^	β	ΔR^2^	β
Step 1	.079		Step 1	.079		Step 1	.079		Step 1	.280***		.111	
Age		.083	Age		.083	Age		.083	Age		− .011		− .227
Education		− .138	Education		− .138	Education		− .138	Education		.207		− .080
MMSE		.265	MMSE		.265	MMSE		.265	MMSE		.388*		.194
Step 2	.106*		Step 2	.081*		Step 2	.079*		Step 2	.035		.097*	
Age		.139	Age		.142	Age		.076	Age		− .003		− .213
Education		− .203	Education		− .150	Education		− .149	Education		− .139		− .096
MMSE		.196	MMSE		.252	MMSE		.242	MMSE		.385***		.148
CPITN 1 sextants		.348*	Mean CPITN		− .292*	Highest CPITN		− .282*	Sum of symptoms		− .190		− .316*
Total R^2^	.184*		Total R^2^	.159*		Total R^2^	157*		Total R^2^	.315***		.208*	

^1^Analyses include only those mind-wandering measures that were significantly related with CPITN scores.

^2^Spontaneous task-unrelated thoughts that were picture-unrelated and present-oriented.

^3^Spontaneous task-unrelated thoughts that were picture-related and past-oriented.

^4^Spontaneous task-unrelated thoughts that were picture-related and past-oriented.

*p* < 0.05, **p < 0.01, ***p < 0.001.

The correlation analyses did not show significant associations between mind-wandering measures and the number of each type of periodontitis pathogen (*p*_s_ > 0.144). However, since the number of pathogens, as a measure of periodontal health, met the criteria under which the dichotomised indicator performs as well as or better than the original continuous indicator^55,56^, for each pathogen we a posteriori assigned participants to two groups: one group with a high number of bacteria (above the median for the sample) and the other group with a low number of bacteria (below the median for the sample). The groups were then compared on mind-wandering measures. Significant group differences were found for *Tannerella forsythia,* which was one of the most common pathogens in our sample. In line with our predictions, individuals with a high number of bacteria showed less mind-wandering that was picture-related and past-oriented (M = 3.60, SD = 4.02) than those with a low number of bacteria (M = 6.43, SD = 5.53) (*t* = − 1.754; *p* < 0.05; *d* = 0.453). Similarly, individuals with a high number of bacteria showed less mind-wandering that was picture-related and future-oriented (M = 1.23, SD = 1.59) than those with a low number of bacteria (M = 2.33, SD = 3.04) (*t* = − 2.271; *p* < 0.05; *d* = 0.586). The cutoff number of *Tannerella forsythia* for group assignment was 15,500, and the groups did not differ in age, education, or gender (*p*_s_ > 0.170).

### Periodontitis status and episodic memory

The periodontitis status measures were not significantly associated with performance in Addenbrooke’s Cognitive Examination III (ACE-III) (see Table 4), neither with performance in several subtests that evaluated attention, fluency, visuospatial abilities and language, nor with the total ACE-III score, from which the memory subtest was excluded (CPITN scores: *p*_s_ > 0.080; number of symptoms subjectively: *p*_s_ > 0.152; number of pathogens *p*_s_ > 0.100). This pattern was in line with our predictions.

**Table 4:** Testing the relationship between poorer periodontal health and poorer episodic memory with zero-order correlations between the Community Periodontal Index of Treatment Needs (CPITN) scores and neuropsychological tests.

	CPITN					
	Sum of sextants with:				Mean CPITN^1^	Highest CPITN^2^
	CPITN 1	CPITN 2	CPITN 3	CPITN 4		
MMSE	.261*	− .047	− .001	− .028	− .100	− .082
Addenbrooke’s cognitive examination (ACE− III)						
Attention	.096	.021	.023	.03	.042	.129
Fluency	.15	− .069	.06	.088	.067	.166
Visuospatial functions	.058	− .063	− .101	.236	.07	.152
Language	.121	.104	.081	.194	− .013	.134
Total (excluding memory)	.182	− .042	.047	.165	.079	.228
California verbal learning test (CVLT)						
Trial 5: free recall	.168	.056	.038	− .279*	− .17	− .135
Trial 5: intrusion errors	− .103	− .063	− .136	.440***	.228	.112
Trials 1–5: free recall	.215	.106	.022	− .256*	− .234	− .145
Trials 1–5: intrusion errors	− .128	− .135	− .097	.268*	.280*	.197
Short delay free recall	.185	.101	− .025	− .185	− .24	− .14
Short delay free recall intrusion errors	− .174	.267*	.143	− .074	− .058	− .058
Long delay free recall	.252	.151	.000	− .109	− .264*	− .13
Long delay free recall intrusion errors	− .062	− .11	− .159	.105	.184	.196

Values that remained statistical significant after Benjamini–Hochberg correction in bold.

^1^Sum of CPITN divided by number of valid sextants.

^2^Highest CPITN among valid sextants; For the number of sextants CPITN 1–2, higher score indicates better gingival health; For the rest of CPITN indices, higher score indicates poorer gingival health; For the CVLT recall, higher score indicates better memory; For the CVLT intrusion errors, higher score indicates worse memory.

*p < .05, **p < .01, ***p < .001.

There was a significant correlation between one measure of periodontitis status (the number of CPITN 1 sextants) and another test measuring general cognitive function, namely MMSE (*r* = 0.261; *p* < 0.05; Table 4). However, it did not contradict our predictions since MMSE includes episodic memory tasks. Furthermore, the relationship between the CPITN 1 sextants and MMSE did not remain significant after adjustment for age and education in a two-step hierarchical regression analysis, with MMSE as a dependent variable and the CPITN 1 sextants as a predictor.

#### The CPITN scores and the California verbal learning test (CVLT)

Four significant correlations were found between the number of sextants with CPITN 4, which are the sextans most severely affected by periodontitis, and the CVLT indices (see Table 4). In line with our predictions, these relationships were negative for recall measures, and positive for the number of intrusion errors. Furthermore, and also in line with our predictions, the mean CPITN was positively associated with the number of intrusion errors from five learning trials, and negatively associated with long delay free recall. Except for a moderate correlation between the CPITN 4 sextants and the number of intrusion errors in the fifth trial, all of these correlations were small.

Table 5 shows the results of hierarchical regression analyses predicting episodic memory performance from background variables (age, years of education, and MMSE scores) and CPITN scores. For all but one of the CVLT indices that were significantly associated with the CPITN 4 sextants in the correlation analyses, the number of CPITN 4 sextants remained a significant predictor: free recall in the fifth trial, *β* = 0.254; *p* < 0.05; intrusions in the fifth trial, *β* = 0.428; *p* < 0.001; intrusions in trials 1–5, *β* = 0.276; *p* < 0.05. Adding the number of CPITN 4 sextants as a second step in the regression model contributed significantly for all these episodic memory measures (see Table 5). In contrast, mean CPTIN was no longer related to episodic memory, for any of the two CVLT indices that were significantly associated with it in the correlation analyses.

**Table 5:** Hierarchical multiple regression analyses predicting California Verbal Learning Test (CVLT) scores from age, education, MMSE scores and the Community Periodontal Index of Treatment Needs (CPITN) scores^1^.

	CVLT Trial 5: Free recall		CVLT Trial 5: Intrusion errors		CVLT Trials 1–5: Free recall		CVLT Trials 1–5: Intrusion errors			CVLT Long delay free recall		CVLT Trials 1–5: Intrusion errors	
	ΔR^2^	β	ΔR^2^	β	ΔR^2^	β	ΔR^2^	β		ΔR^2^	β	ΔR^2^	β
Step 1	.165*		.043		.294***		.027		Step 1	.220**		.027	
Age		− .014		.124		− .163		.146	Age		− .109		.146
Education		− .121		.135		− .177		.004	Education		.031		,004
MMSE		.398**		− .094		.469***		− .049	MMSE		.424**		− .049
Step 2	.062*		.174***		.043		.073*		Step 2	.040		.062	
Age		− .009		.115		− .159		.140	Age		− .067		.094
Education		− .066		.042		− .131		− .056	Education		.023		.014
MMSE		.386**		− .074		.459***		− .036	MMSE		.415**		− .038
CPITN 4		− .254*		.428***		− .213		.276*	Mean CPITN		− .206		.256
Total R^2^	.227**		.217**		.337***		.100		Total R^2^	.260**		.089	

*p < 0.05, **p < 0.01, ***p < *0.001.*

^1^Analyses include only those episodic memory measures that were significantly related to CPITN scores.

#### Periodontitis symptoms, subjectively evaluated, and CVLT

The number of symptoms chosen by the participant on the list of symptoms was not significantly associated with any of the episodic memory test indices (*p*_s_ > 0.072).

#### The number of periodontitis pathogens and CVLT

Seven significant associations were found between the number of different types of pathogen and CVLT indices (see Table 6). In line with our predictions, these relationships were negative for recall measures, and positive for the number of intrusion errors. Most of the correlations were moderate, except for one small correlation between free recall in trials 1–5 and *Tannerella forsythia* and one large correlation between the fifth trial intrusions and the number of *Fusobacterium nucleatum.*

**Table 6:** Testing the relationship between poorer periodontal health and poorer episodic memory with zero-order correlations between the number of detected pathogens per specie and neuropsychological tests.

	Number of pathogens from specie:					
	Porphyromonas gingivalis	Treponema denticola	Tannerella forsythia	Peptostrep. (Micromonas) micros	Fusobacterium nucleatum	Capnocytophaga gingivalis
MMSE	− 0.223	− 0.134	− 0.158	0.069	− 0.058	0.061
Addenbrooke’s cognitive examination (ACE− III)						
Attention	− 0.202	− 0.015	− 0.206	0.018	− 0.115	− 0.057
Fluency	− 0.125	− 0.099	− 0.091	0.090	− 0.105	− 0.045
Visuospatial functions	0.088	0.034	0.146	0.139	0.025	0.115
Language	− 0.070	− 0.008	0.042	0.126	− 0.051	− 0.215
Total (excluding memory)	− 0.155	− 0.076	− 0.096	0.129	− 0.122	− 0.067
California verbal learning test (CVLT)						
Trial 5: free recall	− 0.055	− 0.305*	− 0.21	− 0.193	− 0.168	0.052
Trial 5: intrusion errors	0.135	− 0.069	0.484***	0.340**	0.765***	− 0.059
Trials 1–5: free recall	− 0.092	− 0.238	− 0.265*	− 0.232	− 0.193	0.051
Trials 1–5: intrusion errors	0.009	0.090	0.214	0.306*	0.300*	0.163
Short delay free recall	− 0.260*	− 0.192	− 0.242	− 0.062	− 0.18	0.091
Short delay free recall intrusion errors	− 0.169	0.198	− 0.123	− 0.021	0.014	− 0.084
Long delay free recall	− 0.155	− 0.213	− 0.159	− 0.024	− 0.126	0.028
Long delay free recall intrusion errors	− 0.016	− 0.007	− 0.042	− 0.011	− 0.092	− 0.043

Values that remained statistical significant after Benjamini–Hochberg correction in bold.

For the CVLT recall, higher score indicates better memory; For the CVLT intrusion errors, higher score indicates worse memory.

*p < .05, **p < .01, ***p < .001.

Table 7 shows the results of hierarchical regression analyses predicting episodic memory performance from background variables (age, years of education, and MMSE scores) and the number of bacteria. Three pathogens remained significantly associated with the fifth trial intrusions: *Tannerella forsythia*, *β* = 0.475; *p* < 0.001; *Peptostrep. (Micromonas) micros*, *β* = 0.329; *p* < 0.05; *Fusobacterium nucleatum*, *β* = 0.755; *p* < 0.001, and the addition of the number of these pathogens as a second step in the regression model contributed significantly (see Table 7). Two pathogens remained significantly related to intrusions in trials 1–5: *Peptostrep. (Micromonas) micros, β* = 0.300; *p* < 0.05; *Fusobacterium nucleatum, β* = 0.294; *p* < 0.05, and adding the number of these pathogens as a second step in the regression model contributed significantly (see Table 7). One of the two recall measures that were significantly associated with the number of pathogens in the correlation analyses, namely the recall in the fifth trial, remained significantly related to the pathogens (*Treponema denticola, β* = − 0.276; *p* < 0.05).

**Table 7:** Hierarchical multiple regression analyses predicting California Verbal Learning Test (CVLT) scores from age, education, MMSE score and the number of pathogens^1^.

	CVLT Trial 5: Free recall			CVLT Trial 5: Intrusion errors		CVLT Trials 1–5: Free recall			CVLT Trial 5: Intrusion errors		CVLT Trials 1–5: Intrusion errors			CVLT Trial 5: Intrusion errors		CVLT Trials 1–5: Intrusion errors	
	ΔR^2^	β		ΔR^2^	β	Δ R^2^	β		ΔR^2^	β	ΔR^2^	β		ΔR^2^	β	ΔR^2^	β
Step 1	.165***		Step 1	.043		.294***		Step 1	.043		.027		Step 1	.043		.027	
Age		− .014	Age		.124		− .163	Age		.124		.146	Age		.124		.146
Education		− .121	Education		.135		− .177	Education		.135		.004	Education		.135		.004
MMSE		.398***	MMSE		− .094		.469***	MMSE		− .094		− .049	MMSE		− .094		− .049
Step 2	.073*		Step 2	.217***		.032		Step 2	.103*		.085*		Step 2	.567***		.086*	
Age		.003	Age		.069		− .142	Age		.059		.087	Age		.108		.140
Education		− .152	Education		.147		− .182	Education		.102		− .026	Education		.102		− .009
MMSE		.369***	MMSE		− .034		.446***	MMSE		− .130		− .081	MMSE		− .051		− .032
Td^2^		− .276*	Tf^3^		.475***		− .182	Pm^4^		.329*		.300*	Fn^5^		.755***		.294*
Total R^2^	.238**		Total R	.260**		.326***		Total R^2^	.146		.113		Total R^2^	.610***		.113	

^1^Analyses include only those episodic memory measures that were significantly related to the number of pathogens.

^2^Treponema denticola.

^3^Tannerella forsythia.

^4^Peptostrep. (Micromonas) micros.

^5^Fusobacterium nucleatum.

*p < 0.05, **p < 0.01, ***p < 0.001.

## Discussion

A novel SRD hypothesis argues that individuals in the preclinical stages of AD are particularly impaired in tasks based on spontaneous retrieval, and thus these tasks are sensitive to very early signs of cognitive decline. Our first objective was to provide more evidence to support the SRD hypothesis by showing, for the first time, the relationship between poorer periodontal health, which is considered a risk factor for AD, and reduced spontaneous retrieval, as measured by fewer mind-wandering. Our second objective was to provide evidence that poorer periodontal health is particularly associated with worse episodic memory. Therefore, we expected to show the relationship between periodontitis and performance on the comprehensive episodic memory test, rather than the association between periodontitis and the measure of general cognitive function from which memory was excluded. We confirmed the expected relationships.

### The spontaneous retrieval deficit hypothesis: relationship between periodontitis and mind-wandering

We found several significant associations between measures of mind-wandering and periodontitis, across subjective and objective indices of oral health, and all were in the expected direction. Importantly, all but one of these associations remained significant after adjustment for age, education, and general cognitive function (as measured by MMSE scores). The latter finding, together with the fact that quite a few relationships between periodontitis and the California Verbal Learning Test were no longer significant after adjustment for MMSE scores, supports our argument about the advantage of mind-wandering as an early marker of cognitive decline. Specifically, compared to cognitive processes captured by neuropsychological tests, including the episodic memory test that we used, mind-wandering is less dependent on general cognitive function.

Although correlation analyses did not show significant associations between the number of periodontitis pathogens and mind-wandering, due to median-split we did find fewer mind-wandering, picture-related and oriented either toward the past or future, in the group with an elevated number of *Tannarella forsythia,* which was one of the most common pathogens in our sample. Given the exploratory nature of these findings, they should be interpreted with caution. However, it should be noted that they are in agreement with the results of the correlation analyses between mind-wandering and other periodontitis measures. They are also in line with previous studies in which individuals with aMCI had spontaneous retrieval deficits primarily within the same two types of mind-wandering: stimulus-related thoughts that were oriented either toward the past or future^28,29^.

Taken together, our findings significantly expand previous data on reduced mind-wandering in aMCI and early stages of AD, and provide novel evidence to support the SRD hypothesis by showing the relationship between spontaneous retrieval and the risk factor of AD, namely periodontitis.

### Relationship between periodontitis and episodic memory

We found many significant associations between episodic memory indices and periodontitis status, objectively measured by both CPITN and pathogens, and a substantial part of them remained significant after adjustment for age, education, and general cognitive function. All associations were in the expected direction, and they were stronger for the number of pathogens than CPITN. At the same time, no relationship was found between periodontitis and the tests measuring specific cognitive abilities, other than memory, or a general index of cognitive function (Addenbrooke’s Cognitive Examination III) from which memory was excluded. These findings unequivocally support the claim that periodontal health is particularly related to episodic memory, and may help to gain a clearer understanding of the association between oral health and dementia.

A particular link between periodontitis and memory suggests that periodontal health may be primarily related to an elevated risk of Alzheimer’s type dementia, the early stages of which, unlike other types of dementia, manifest with memory impairment^49–51^. This conclusion is supported by the results of biomolecular studies that have shown associations between periodontitis and specifically AD, e.g., the presence of periodontitis pathogens within brain tissue from individuals with AD in post-mortem assessment^57^; the presence of periodontitis bacteria’s DNA in cerebrospinal fluid from individuals with probable AD^38^; a decreased ability to learn and memorise following intracellular accumulation of β-amyloid after chronic exposure to the periodontitis pathogen that was demonstrated in the animal model^58^, and increased production of β-amyloid and tau protein in mice’ brain, which is AD-specific pathology, after chronic oral exposure to periodontitis pathogen^59^.

Our results thus expand the accumulating data that suggest that periodontitis is primarily related to the elevated risk of AD, by showing its relationship with episodic memory in general and spontaneous retrieval in particular. It has important implications for research on early identification of AD risk, as well as clinical practise. The data suggest that the presence and severity of periodontitis should be considered when projecting the probability of progression to AD in preclinical groups or when developing questionnaires and clinical inventories designed to assess such risk. Regarding clinical practise, they show the importance of taking special care of gingival health in individuals with an elevated likelihood of progression to AD as a means of reducing the risk of progression.

### Limitations and future directions

Despite encouraging findings, the present study has some limitations that will need to be addressed in future research, such as weak to moderate associations between periodontitis status and mind-wandering, and periodontitis status and episodic memory. Furthermore, these associations were found only for some measures of mind-wandering and episodic memory. The lack of stronger associations may be due to the characteristics of the sample that consisted of high functioning, well educated, and community-dwelling older adults with a restricted range of periodontal health indices. Since participants were able to take care of their dental health, the sample did not include many of those with highly developed periodontal disease. This explanation is in line with the results of previous studies suggesting that the relationship between oral health and cognitive function is stronger for groups with a lower overall, and of a wider range, oral health status (e.g., residents of nursing homes), compared to high functioning, community-dwelling older adults^34,60^. To ensure a greater variance in periodontitis status, future studies on the relationship between periodontitis and specific memory processes can recruit both high functioning community-dwelling adults and residents of nursing homes.

It should be noted that the pattern of our results suggests that mind-wandering is more consistently associated with CPITN scores and subjective evaluation of periodontal symptoms, while episodic memory is more consistently associated with the number of pathogens and CPITN scores, with the majority of the associations found with the number of sextants most severely affected by the disease (CPITN 4). It may be due to the fact that the three measures of oral health applied in our study provide somewhat different types of information on oral health status. The number of bacteria represents the current scale of infection with certain types of periodontitis pathogens, while CPITN describes the visible changes in the structure of the gums caused by periodontitis over the years. These changes are caused by the gingivitis infection, but can remain observable even after the gingivitis infection is treated or decreased^61^. Therefore, it is possible to have a low number of periodontitis bacteria due to the applied gingivitis treatment and, at the same time, to have visible moderate changes in gum structure caused by periodontitis over the years. Similarly, the chosen symptoms show what kind of periodontitis symptoms the participant has experienced during the course of the disease, even if they do not have gingivitis or an elevated number of bacteria at the time of examination, due to the previously applied treatment. This reasoning is supported by our additional analyses in which a significant relationship with the number of bacteria was found for the number of CPITN 4 sextants, but not for the other three CPITN codes or the number of symptoms. Therefore, the pattern of relationships may suggest that mind-wandering is more related to cumulative, but not very severe, changes caused by well-managed disease over the years, while episodic memory is more related to the most severe changes in the gums caused by poorly managed periodontitis. Future studies may address this issue more directly.

Finally, since our investigation was a single-assessment cross-sectional study, further longitudinal examination is needed to be able to unequivocally determine the causality and directionality behind the relationships that we demonstrated. Of particular interest would be the use of prospective longitudinal studies to investigate how simple tasks relying on spontaneous retrieval will compare with standard neuropsychological tests currently used, in terms of early detection of MCI and prediction of conversion rates to AD. Future studies may also investigate the relationship between deficits in spontaneous cognitions and biological markers of AD (e.g., amyloid plaques or the ApoE4 gene).

## Method

### Participants

A total of 60 participants (M_age_ = 72.52; SD = 4.15; 86% women) who lived independently in the community, with varying periodontal treatment needs were recruited. To ensure sufficient power, we performed the a priori power analysis on GPOWER 3.1^62^. Since there were no published studies on the relationship between periodontal health and spontaneous retrieval, as measured by mind-wandering, the calculation of the effect size was based on the relationship found by Manchery et al.^34^ (*r* = − 0.51) between periodontal health and an event-based prospective memory task. According to theoretical explanations of prospective memory^36,37^, performance on event-based prospective memory tasks, in contrast to performance on the other type of prospective memory tasks (time-based), can be based on spontaneous retrieval of intended actions. With an alpha level of 0.05 and a minimum power of 0.95, 39 participants were necessary to find a statistically significant effect for a zero-order correlation. However, Manchery et al.^34^ found the relationship between objectively measured periodontal health and prospective memory among older adults living in a retirement village, and suggested that this link may be weaker among older adults who live independently in the community, among which oral health is generally much better, so the range of oral health indexes is relatively restricted. Therefore, to avoid the risk of not having enough power to capture the relationship between oral health and mind-wandering, we recruited more participants than the calculations indicated were necessary.

The participants were members of senior social clubs or volunteers in the community, and they all received 150 PLN (approximately 34 USD) for their participation. The study was carried out in accordance with the Declaration of Helsinki and was approved by the Bioethics Research Committee at Jagiellonian University (Opinion number: 1072.6120.76.2022). Participants provided their informed written consent to take part in the study. For all participants, inclusion criteria were: (a) no head/brain injuries, (b) no history of cerebrovascular disease, (c) no current dependence on alcohol or substances, (d) no medical, neurological, or psychiatric disorders resulting in cognitive dysfunctions, (e) age more than 65 years, (f) not meeting the criteria of the Diagnostic and Statistical Manual of Mental Disorders’ (DSM-5) for dementia^63^, (g) preserved general cognitive function as confirmed by a normal score on the Mini-Mental State Examination (MMSE)^64^ (normality cut-off score: 24)^65^—MMSE scores in the sample ranged from 25 to 30, (h) maintained activities of daily living as confirmed by a maximum score on the Instrumental Activities of Daily Living (IADL) subscale of the Nurses’ Observation Scale for Geriatric Patients (NOSGER)^66,67^ (i) absence of severe depression, as confirmed by a score below 10 on the Geriatric Depression Scale 15^68^ . Fluency in Polish and adequate vision and hearing were also required. Inclusion criteria were evaluated in the initial interview screening.

### Measures

#### Neuropsychological evaluation

A Polish version of the Mini-Mental State Examination^64^ was used in the initial screening for dementia. To measure episodic memory, a Polish version^69^ of the California verbal learning test (CVLT)^52^ was used. During CVLT, the experimenter reads a list of 16 nouns aloud over five learning trials. After each trial, the participant is asked to recall as many words as possible. After the fifth trial, a distractor list, with new 16 words, is presented. Free recall of the original list is tested immediately (short delay), and again after 20 min (long delay). We calculated four free recall measures for which a higher score indicated better performance: (a) the number of words recalled after the fifth trial, (b) the number of words recalled across all five trials, (c) the number of words recalled after the short delay, and (d) the number of words recalled after the long delay. Furthermore, the number of intrusion errors (recalling words that were not present on the original list) was calculated after the fifth trial, for all five trials together, after the short delay and the long delay, with a higher score indicating poorer performance. Attention, executive functions, language and visuospatial abilities were tested with Addenbrooke’s Cognitive Examination III (ACE-III)^70^. There were three tasks in the attention subscale (maximum score—18), two verbal fluency tasks in the executive function subscale (max. 14), seven tasks in the language subscale (max. 26), and five tasks in the visuospatial abilities subscale (max. 16). For all these subscales, a higher score indicated better performance. ACE-III also included five memory tasks, but the memory subscale was excluded from the total ACE-III score.

#### Oral health evaluation

An eight-item list of warning signs of periodontal disease was developed. The list of symptoms was based on^71^ and symptoms published by the Centres for Disease Control and Prevention of the United States^72^. Participants were asked to confirm or deny experiencing any of the symptoms on the list (YES/NO answer). The list included: (a) swollen gums, (b) redness of the gums, (c) bleeding gums during brushing or spontaneously, (d) exposure of tooth necks, (e) migration of teeth, (f) loosening of teeth, (g) sore gums while brushing, (h) unpleasant smell from the mouth, (i) unpleasant taste in the mouth, (j) recurrent gingivitis, and (k) hypersensitivity of teeth to extreme temperatures.

To capture possible associations between gingival health and cognitive functioning rather than diagnose participants with periodontitis, we measured the number and severity of symptoms of periodontal disease. Two types of oral examination were performed in one specialist dental clinic by the same single examiner who was a qualified dentist and had no access to the data about the participants collected during the neuropsychological evaluation session. As the first type of oral examination, gingival health was evaluated using the community periodontal index of treatment needs (CPITN) recommended by the WHO^73^. The index could range from 0 to 4, with: 0 for healthy periodontium, 1 for gingival bleeding on probing (gingivitis infection), 2 for the presence of subgingival calculus (i.e., 0–2 indicated no symptoms of periodontitis), 3 for the presence of at least one pathological gingival pocket 4–5 mm (symptom of periodontitis), and 4 for at least one pathological gingival pocket 6 mm or more (symptom of severe periodontitis) indicating the need for complex treatment^74^. The index was calculated for each tooth sextant as the tooth score with the highest CPITN. Sextants with less than 2 teeth were excluded from the calculations. We analysed CPITN scores at various levels. First, we analysed the number of sextants with each CPITN code per participant. Since CPITN codes 3 to 4 indicate the presence of pathological gingival pockets, i.e., sextants considered to be severely affected by the disease^75^, the higher the number of sextans with these CPITN codes, the poorer the gingival health. In contrast, CPITN codes 0–2 indicate a lack of pathological gingival pockets, and therefore, the higher the number of sextans with these CPTN codes, the better the gingival health. Second, for each participant, we calculated the mean CPITN score (i.e., the sum of CPITN codes divided by the number of valid sextants), and the highest CPITN score. For both indices, a higher score indicated poorer gingival health.

As the second type of oral examination, the number and type of periodontitis pathogens present within the periodontium were examined with PET plus (MIP Pharma®, Germany). During the examination procedure, the dentist placed, for 20 s, a special sterile dental filter in the periodontal pockets, or in the area of the periodontium, if the participant was healthy. One pooled sample per participant was collected from four sextants with the deepest pathological gingival pockets. If pocket depths did not exceed 3 mm in the individual (which was the case only for 4 participants), the sample was taken from four different sextants with the deepest pockets within the normal limit (i.e., < 3 mm). If there were fewer than four sextants present, the sample was taken four times from the available sextants. After that, the filter was secured in a special transport sample, and then sent to the MIP Pharma® specialistic biomolecular laboratory in Germany by ordinary mail. In the laboratory, the sample was analysed using the real-time polymerase chain reaction (PCR) method to isolate the DNA of bacteria. The test was designed to detect nine types of periodontitis bacteria: *Aggregatibacter actinomycetencomitans, Porphyromonas gingivalis, Treponema denticola, Tannerella forsythia, Prevotella intermedia, Peptostreptococcus micros, Fusobacterium nucleatum, Eubacterium nodatum, and Capnocytophaga gingivalis,* which are the most prevalent periodontitis pathogens^76–79^. We analysed the number of detected pathogens for each participant, separately for each type of bacteria.

#### Mind-wandering evaluation

Participants completed a computer-based Man-made/Natural Task, which was originally developed by Maillet and Schacter^5^, and then modified and used to test the SRD hypothesis by Wereszczyński and Niedźwieńska^29^. The task consisted of a 242-slide presentation of pictures showing natural objects (e.g., flower) and man-made objects (e.g., car). Below each picture was a caption corresponding to it**.** Participants were asked to decide whether the object depicted was artificial or natural. Each stimulus was presented for 4 s, followed by a blank screen for 4 s. Every 6–10 stimulus slides, the task stopped and thought probe questions appeared on the screen. Participants were asked to describe their thought content the moment before the question appeared on the screen by choosing one of the following answers: (1) I didn’t have any thoughts; (2) I had a thought triggered by one of the pictures I saw; (3) I had a thought unrelated to the task or any of the pictures I saw; (4) I was thinking how I feel about doing this task. It is important to note that the last category (task-related thoughts) would predominantly include so called task-related interference^5,80^, i.e., concerns about task performance (e.g., *Oh no! I’ve chosen wrong answer!*) or opinions about the task itself (e.g., *This task is very easy*). Such thoughts may sometimes include references to the pictures, but only to the pictures as parts of the task, and they will still be expressing, for example, concerns about the task performance (e.g., *The tree is natural but I have chosen that it’s man-made!*) or opinions about the task itself (e.g., *Pictures take too long to change*). In contrast, thoughts from the second category (picture-related thoughts) would be direct associations with the pictures, without any reference to the task, e.g., *My friend is a mechanic* after seeing the picture of a car.

If participants had stimuli-related thoughts, they were additionally asked which picture had triggered the thought. The participants were then asked whether the thought they had was spontaneous or deliberate. Finally, they were asked if the thought they had was about the past, present, or future. The categories of thoughts and the thought probing procedure were adapted from Maillet and Schacter^5^ and Wereszczyński and Niedźwieńska^29^ (see also^28,81^ for similar thought probing).

The presentation of the stimuli and the response collection were controlled by Inquisit 5 software running on a 14″ foldable notebook. Pictures measured on average 600 px (height) × 600 px (width) at a viewing distance of 60 cm, and were presented on a white background in the centre of the screen. They were generated in random order, which was then the same for each participant. Since older Polish adults may not be very familiar with using the computer, all participants gave their answers orally, rather than typing them into the computer, and the experimenter manually recorded the responses of the participants.

Stimuli-pictures were obtained from the same base as used by Maillet and Schacter^5^, that is, the Bank of Standardised Stimuli^82,83^. The base consists of stimuli that were assessed on different dimensions by a large number of participants, as part of normalisation studies. One of these dimensions was familiarity, measured by the question: “Rate the level to which you are familiar with the object” on a 5-point scale (1 = very unfamiliar; 5 = very familiar). For the study by Wereszczyński and Niedźwieńska^29^, 300 pictures with the highest familiarity scores and 300 pictures with the lowest familiarity scores were chosen from the base, and then piloted among older Polish adults to obtain those that would be most familiar and most unfamiliar for a Polish sample. Since Wereszczyński and Niedźwieńska^29^ showed that mind-wandering was much more likely when older adults were exposed to highly familiar pictures, compared to when they were exposed to highly unfamiliar pictures, a total of 242 pictures with the highest mean familiarity (M = 4.16; SD = 0.34) were selected for the final set in the present study. Due to the predominance of pictures showing man-made objects among the pictures rated as most familiar, pictures with natural objects accounted for 1/3 of the stimuli.

As should be with a cognitive task, during which mind-wandering is evaluated, the performance of the participants on the Man-made/Natural Task, as measured by the percentage of correct answers out of all answers provided, was at ceiling (M = 96.45, SD = 2.19). The performance was not associated with any of the mind-wandering measures (*p*_*s*_ > 0.223).

### Procedure

Participants were individually tested in two psychological evaluation sessions (each approximately 1 h long) on separate days, and in one dental examination session (approximately 0.5 h long) which took place between psychological evaluation sessions. The screening interview, NOSGER-IADL, MMSE, ACE-III, and the Geriatric Depression Scale 15 were administered at the first psychological evaluation session. The Man-made/Natural Task and CVLT were completed in the second.

At the beginning of the second psychological evaluation session, participants completed short-delay CVLT tasks. They were then briefly introduced to the Man-made/Natural Task. The procedure of administering the task was the same as in Wereszczyński and Niedźwieńska^29^, and its description is based on their paper. Participants were asked to press ‘S’ on the keyboard if the object on the screen was man-made, and to press ‘N’ if it was natural. They were also informed that we are interested in what types of thoughts people experience while performing such tasks. Therefore, the slide presentation would occasionally stop, at which point they would be prompted to report their thoughts at the exact moment they were stopped. Participants were briefly informed about the thoughts they might experience during the task and what options they would have to categorise them, i.e., no thoughts, picture-triggered off-task thoughts, picture-unrelated off-task thoughts, and thoughts about the experience of performing the task. This was followed by training, during which participants were given examples of thoughts of various categories and asked what category they would choose. If they made the wrong choice, it was explained why it should be a different category. The exemplary thoughts were, among others: *I used to work as a bus driver* after seeing the picture of a bus; *I have a doctor appointment tomorrow*, with no picture related to this fact; *I wonder if I have chosen the right answer*. The training continued until the participant was able to correctly categorise all types of thoughts. The participants were then explained the difference between spontaneous thoughts (thoughts that pop into mind without your intention) and deliberate thoughts (something you deliberately chose to think about). Finally, participants were briefly informed about the types of off-task thinking they could experience, i.e., that it could be related to something that: (a) was happening in the present, at any point in the course of the task (e.g., *I love my family*); (b) had happened in the past, before starting the task (e.g., *I went to Spain last year*); (c) would happen in the future, after completing the task (e.g., *I am going to eat delicious supper today*). This was followed by a short practise with two 10-slide trials and two thought probes. After practising, participants completed the long-delay CVLT tasks and the Man-made/Natural Task.

## Data Availability

The data used to support the findings of this study are available from the corresponding author upon request.
